# Rapid diagnostic tests failing to detect infections by *Plasmodium falciparum* encoding *pfhrp2* and *pfhrp3* genes in a non-endemic setting

**DOI:** 10.1186/s12936-020-03251-3

**Published:** 2020-05-11

**Authors:** Grégoire Pasquier, Vincent Azoury, Milène Sasso, Laëtitia Laroche, Emmanuelle Varlet-Marie, Sandrine Houzé, Laurence Lachaud, Patrick Bastien, Yvon Sterkers, Maude F. Leveque

**Affiliations:** 1 University of Montpellier, CNRS, IRD, UMR MiVEGEC, Montpellier, France; 2 Department of Parasitology-Mycology, CHU de Montpellier, Montpellier, France; 3 Laboratory of Microbiology, CHU de Nîmes, Nîmes, France; 4 Centre National de Référence du Paludisme, APHP, Hôpital Bichat-Claude Bernard, Paris, France

**Keywords:** False-negative RDTs, Imported malaria, Gene deletion, Genetic diversity, LAMP, Screening, Low parasitemia, Microscopy, Misdiagnosis

## Abstract

**Background:**

Rapid diagnostic tests (RDTs) detecting the histidine-rich protein 2 (PfHRP2) have a central position for the management of *Plasmodium falciparum* infections. Yet, variable detection of certain targeted motifs, low parasitaemia, but also deletion of *pfhrp2* gene or its homologue *pfhrp3,* may result in false-negative RDT leading to misdiagnosis and delayed treatment. This study aimed at investigating the prevalence, and understanding the possible causes, of *P. falciparum* RDT-negative infections at Montpellier Academic Hospital, France.

**Methods:**

The prevalence of falsely-negative RDT results reported before and after the introduction of a loop-mediated isothermal amplification (LAMP) assay, as part as the malaria screening strategy in January 2017, was analysed. Negative *P. falciparum* RDT infections were screened for *pfhrp2* or *pfhrp3* deletion; and exons 2 were sequenced to show a putative genetic diversity impairing PfHRP2 detection.

**Results:**

The overall prevalence of *P. falciparum* negative RDTs from January 2006 to December 2018 was low (3/446). Whereas no cases were reported from 2006 to 2016 (0/373), period during which the malaria diagnostic screen was based on microscopy and RDT, prevalence increased up to 4.1% (3/73) between 2017 and 2018, when molecular detection was implemented for primary screening. Neither *pfhrp2/3* deletion nor major variation in the frequency of repetitive epitopes could explain these false-negative RDT results.

**Conclusion:**

This paper demonstrates the presence of *pfhrp2* and *pfhrp3* genes in three *P. falciparum* RDT-negative infections and reviews the possible reasons for non-detection of HRP2/3 antigens in a non-endemic setting. It highlights the emergence of falsely negative rapid diagnostic tests in a non-endemic setting and draws attention on the risk of missing malaria cases with low parasitaemia infections using the RDT plus microscopy-based strategy currently recommended by French authorities. The relevance of a novel diagnostic scheme based upon a LAMP assay is discussed.

## Background

Malaria remains a major public health issue in tropical regions and accounts for a significant burden in non-endemic areas. Imported malaria indeed represents one of the most prevalent infectious diseases among travelers and migrants in industrialized countries, France being the most impacted European country [1]. A cross-sectional study of 43,333 French malaria cases reported an 85% rate of *Plasmodium falciparum* infection and a significant increase in severe cases between 1996 and 2016 [2]. Accurate diagnosis and prompt treatment are essential to prevent life-threatening complications mostly caused by *P. falciparum* [3]. Biological screening procedures include light microscopy, immunochromatography or molecular techniques used either individually or in combination.

Microscopic diagnosis, by examination of Giemsa-stained thin and thick blood smears, remains the standard method to identify and quantify *Plasmodium* parasites but may be time-consuming for initial screening, especially in non-endemic countries where samples are often negative, and relies upon highly trained personnel [4]. On the other hand, rapid diagnostic tests (RDTs) have emerged as a safe, easy to perform, alternative to microscopy [5]. Most RDTs target repetitive epitopes specific to *P. falciparum* which are encoded by an abundant secreted antigen, the histidine-rich protein 2 (PfHRP2). Its homologue, PfHRP3, shares significant sequence homologies and, as such, may be recognized by monoclonal antibodies raised against PfHRP2 [6]. Although diagnostic performances vary greatly between brands [7], PfHRP2-based RDTs generally display higher sensitivities and specificities for the diagnosis of *P. falciparum* infections than those targeting pan-*Plasmodium* aldolase or lactate dehydrogenase (pLDH) [8, 9]. A growing number of studies yet reported false-negative RDTs results due to partial or complete gene deletion of *pfhrp2* and/or *pfhrp3* (reviewed in [10]). Genetic diversity producing variations in the targeted amino-acid repeats may also affect test performances [6, 11, 12]. Alternative diagnostic approaches include molecular methods which display high sensitivity but are generally technically demanding and time consuming, thus not suitable for urgent diagnosis [13]. In this context, loop-mediated isothermal amplification (LAMP) assays have proven highly effective for rapid *Plasmodium* screening [14–18].

Since 2017, according to the French National Authority for Health [19] and the French Infectious Diseases Society (SPILF) [20], microscopy is still the reference method for initial screening and follow-up and may be combined with RDTs targeting both pan-*Plasmodium* and PfHRP2 antigens. Both techniques were used at the Parasitology-Mycology Department of Montpellier Academic Hospital for all suspected malaria cases until December 2016, when a novel strategy, based upon a LAMP assay and a PfHRP2-based RDT, was introduced for primary diagnosis of malaria. This allowed detecting falsely-negative RDT results, of which the cause was investigated in this study.

## Methods

### Study design

The aim of the study was to investigate the prevalence and possible causes of RDT-negative *P. falciparum* infections over 13 years, from January 2006 to December 2018, when two diagnostic schemes for malaria screening were used. The study population included all cases of *P. falciparum* imported malaria diagnosed at the Parasitology-Mycology Department of Montpellier Academic Hospital, France.

### Malaria diagno**s**tic strategies

**Fig. 1:**
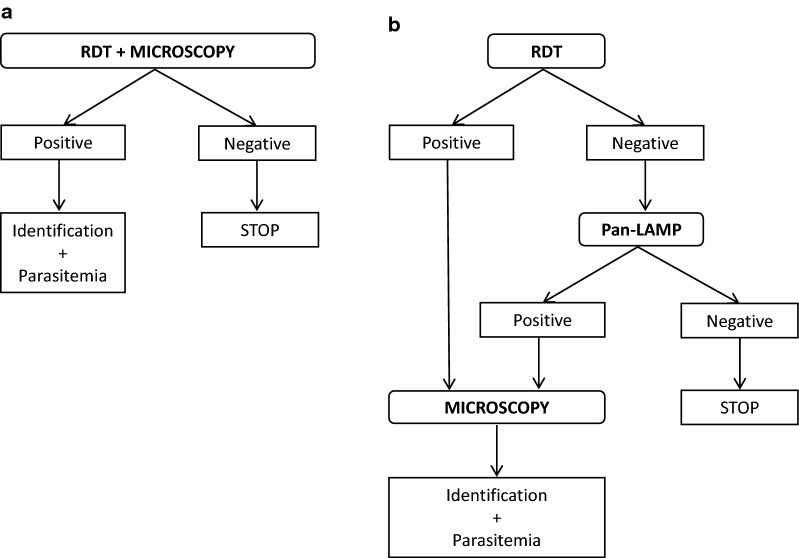
Distinct malaria diagnostic strategies applied from January 2006 to December 2016 (**a**) and from January 2017 to December 2018 (**b**). During the first period (**a**), ICT Malaria Combo Cassette Test (ICT Diagnostics) was used from January 2006 to August 2009 and SD Bioline Malaria Ag Pf/Pan (Standard Diagnostics, Inc.; product code 05FK60), was used from September 2009 to December 2016. During the second period (**b**), SD Bioline Malaria Ag Pf/Pan (Standard Diagnostics, Inc.; product code 05FK60) was continued

Two distinct procedures for screening patients with clinical suspicion of malaria (*i.e*. any febrile patient with a history of travel to malaria-endemic areas), both using antigenic detection by a RDT, were applied in the laboratory (Fig. 1). From January 2006 to December 2016, initial screenings were performed by PfHRP2-based RDTs combined with microscopic examination of thin and thick blood smears (Fig. 1a). In January 2017, a LAMP assay was implemented associated with a RDT, replacing microscopy when both antigenic and molecular tests are negative (Fig. 1b). When either test was positive, thin and thick stained blood films were examined for *Plasmodium* identification and quantification.

### Laboratory procedures

Rapid immunochromatography tests were performed according to manufacturers’ instructions. ICT Malaria Combo Cassette Test (ICT Diagnostics; product code ML02), which targets pan-aldolase and PfHRP2 antigens, was used from January 2006 to August 2009. SD Bioline Malaria Ag Pf/Pan (Standard Diagnostics, Inc.; product code 05FK60), detecting pLDH and PfHRP2 antigens, was used from September 2009 to December 2018.

Thin films were stained with May-Grünwald Giemsa and were examined using oil immersion magnification (1000×) for at least 20 min before being considered negative. Parasites density was estimated as the percentage of infected red blood cells.

Thick smears were stained with Giemsa [21]. All the spot was examined under 1000× magnification. For this study, parasite densities of false negative RDT samples, expressed as the number of parasites/µL, was assessed on thick blood smears and corresponds to the number of parasites per 200 leukocytes, based upon an estimated average of 8000 leukocytes/µL of blood.

For LAMP assays, samples were processed using the Alethia^®^ Malaria kit (Meridian^®^), targeting a pan-*Plasmodium* mitochondrial DNA sequence, according to the manufacturer’s instructions.

### Analysis of false-negative results

All samples with conflicting results (*i.e.* positive pan LAMP and negative RDT) were further analysed for molecular and antigenic testing at the Microbiology Laboratory of Nîmes Academic Hospital (France). Samples were retested 2 days after patient sampling by another RDT used for routine practice: the BinaxNOW RDT (Inverness Medical Innovations, 100 Inc.; product code 660-000) targeting pan aldolase and PfHRP2 antigens. For molecular screening, DNA was extracted from 200µL of whole blood using EZ1^®^ DNA Blood 200 µL kits (QIAGEN^®^) on the Biorobot^®^ EZ1 workstation, according to the manufacturer’s instructions. Two in-house qPCR methods, one distinguishing *Plasmodium* species by specific melting curves of the 18S rRNA [22] and one detecting the *P. falciparum*-specific *cox1* gene [23], were used to confirm malaria infection and *P. falciparum* identification.

### Amplification and sequencing of *pfhrp2* and *pfhrp3*

Samples positive for *P. falciparum* and presenting RDT false-negative results were tested for putative *pfhrp2* and/or *pfhrp3* gene deletion. A *P. falciparum* RDT-positive sample from Gabon diagnosed in January 2019 with a parasitaemia at 0.05% was included for differential analysis of *pfhrp2* and *pfhrp3* sequences compared to those of RDT-falsely negative samples. Genomic sequences of *pfhrp2* (PF3D7_0831800) and *pfhrp3* (PF3D7_1372200) were retrieved from PlasmoDB database (http://www.PlasmoDB.org). Pair of primers specific to the 5′ and 3′ ends of exon 2 of *pfhrp2* (CAAAAGGACTTAATTTAAATAAGAG; AATAAATTTAATGGCGTAGGCA) (expected size: 816 bp) and *pfhrp3* (AAATAAGAGATTATTACACGAAAG; TGGTGTAAGTGATGCGTAGT) (expected size: 698 bp) were used to assess gene deletion following previous recommendations [24]. Amplification of *pfhrp2* and *pfhrp3* was performed using *Pfu*II polymerase (Agilent) under the following cycling conditions: 94 °C for 2 min followed by 35 cycles of 94 °C for 20 s, 54 °C for 20 s, 62 °C for 90 s and 62° for 7 min. PCR products were purified using spin columns (QIAGEN^®^) and sent for Sanger sequencing (Eurofins Genomic^®^). Nucleotide sequences were translated into corresponding amino acids and aligned against the Pf3D7 reference genome using NPS@ (Network Protein Sequence Analysis) software and ESPript 3.0 program for data assembling. Frequency of repetitive histidine and alanine motifs (*i.e.* AHHAHHAAD and AHHAAD) were assessed and sensitivities were predicted according to Baker’s model [11].

## Results

### Detection of *P. falciparum* cases falsely negative for RDT

**Fig. 2:**
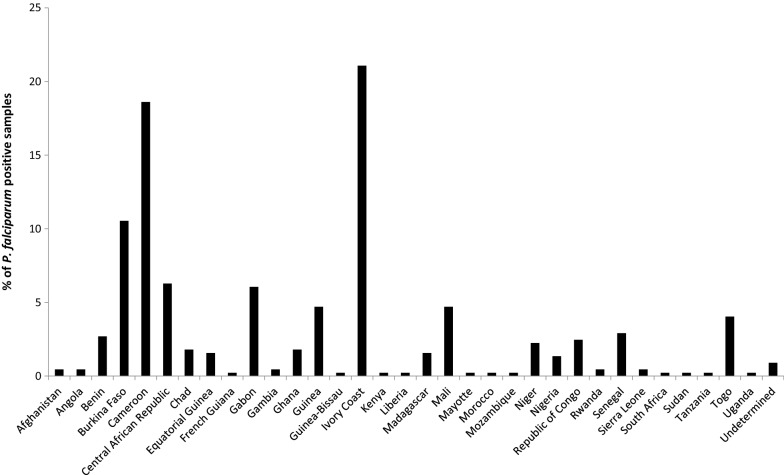
Countries of origin for the 446 *P. falciparum* positive samples reported from January 2006 to December 2018

A total of 446 *P. falciparum* positive samples were diagnosed at the Parasitology Department of the Academic Hospital of Montpellier from January 2006 to December 2018. Almost all patients originated from African countries (Fig. 2). During this period, only three samples with negative RDT results and positive detection by microscopic, LAMP and qPCR assays were detected (Table 1), yielding an overall prevalence of RDT-falsely negative *P. falciparum* infections of 0.67%. In-house qPCR methods were used as the reference to confirm *P. falciparum* infection and exclude possible co-infection with another *Plasmodium* species. Of note, this prevalence was null for the 373 *P. falciparum* infections reported from 2006 to 2016, but raised to 4.1% (over 73 *P. falciparum* infections) between January 2017 and December 2018, *i.e.* after the introduction of the novel scheme for primary diagnosis of malaria. The three patients presented fever with history of recent travelling from endemic countries: Cameroun, Ivory Coast or Gabon in June 2017, August 2018 and December 2018, respectively. Non-detection of PfHRP2 in these three samples was confirmed by two RDTs from different brands (*i.e.* SD Bioline and BinaxNOW). Parasite densities in RDT-negative samples were estimated on thin and thick blood smears and ranged from < 0.001 to 0.05% or ~ 5 parasites/µL to ~ 800 parasites/µL, respectively (Table 1).

**Table 1:** Parasitemiae of the three RDT-negative *P. falciparum* infections in this study

Date	Origin	RDT	LAMP	qPCR	Thin film	Thick film
06-2017	Cameroun	−	+	*Pf*	< 0.001%	~5p/µL
08-2018	Ivory Coast	−	+	*Pf*	< 0.001%	~10p/µL
12-2018	Gabon	−	+	*Pf*	0.05%	~800p/µL

*RDT*, rapid diagnostic test; LAMP, loop-mediated isothermal amplification; qPCR, quantitative polymerase chain reaction; *Pf*, *Plasmodium falciparum*; p, parasites

### Amplification of *pfhrp2* and *pfhrp3* genes

**Fig. 3:**
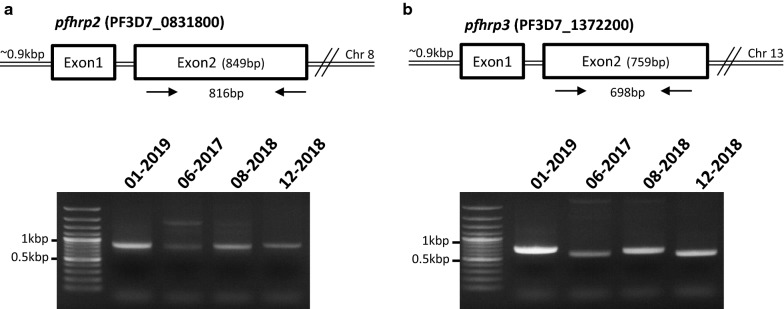
Amplifications of exon2 of *pfhrp2* (**a**) and *pfhrp3* (**b**) from *P. falciparum* negative RDT isolates. First lane: molecular ladder; second lane: positive control; third to fifth lines: false negative RDT isolates

In view of this increase in falsely negative RDTs, and because of the growing numbers of studies reporting strains lacking *pfhrp2* and/or *pfhrp3* genes (reviewed in [10]), *P. falciparum* isolates were tested for putative gene deletion.. Genes encoding PfHRP2 and PfHRP3 are present on chromosome 8 and 13, respectively, with two exons being interrupted by one intron (Fig. 3). Here, using primers specific to exon 2, *pfhrp2* (Fig. 3a) and *pfhrp3* (Fig. 3b) fragments were amplified in the positive control and the three RDT-falsely negative isolates (06/2017; 08/2018; 12/2018), with sizes ranging from 600 to 900 bp, This allowed ruling out *pfhrp2/3* deletion as the cause of these falsely negative RDTs.

### Sequence variations in the *pfhrp2* gene

**Fig. 4:**
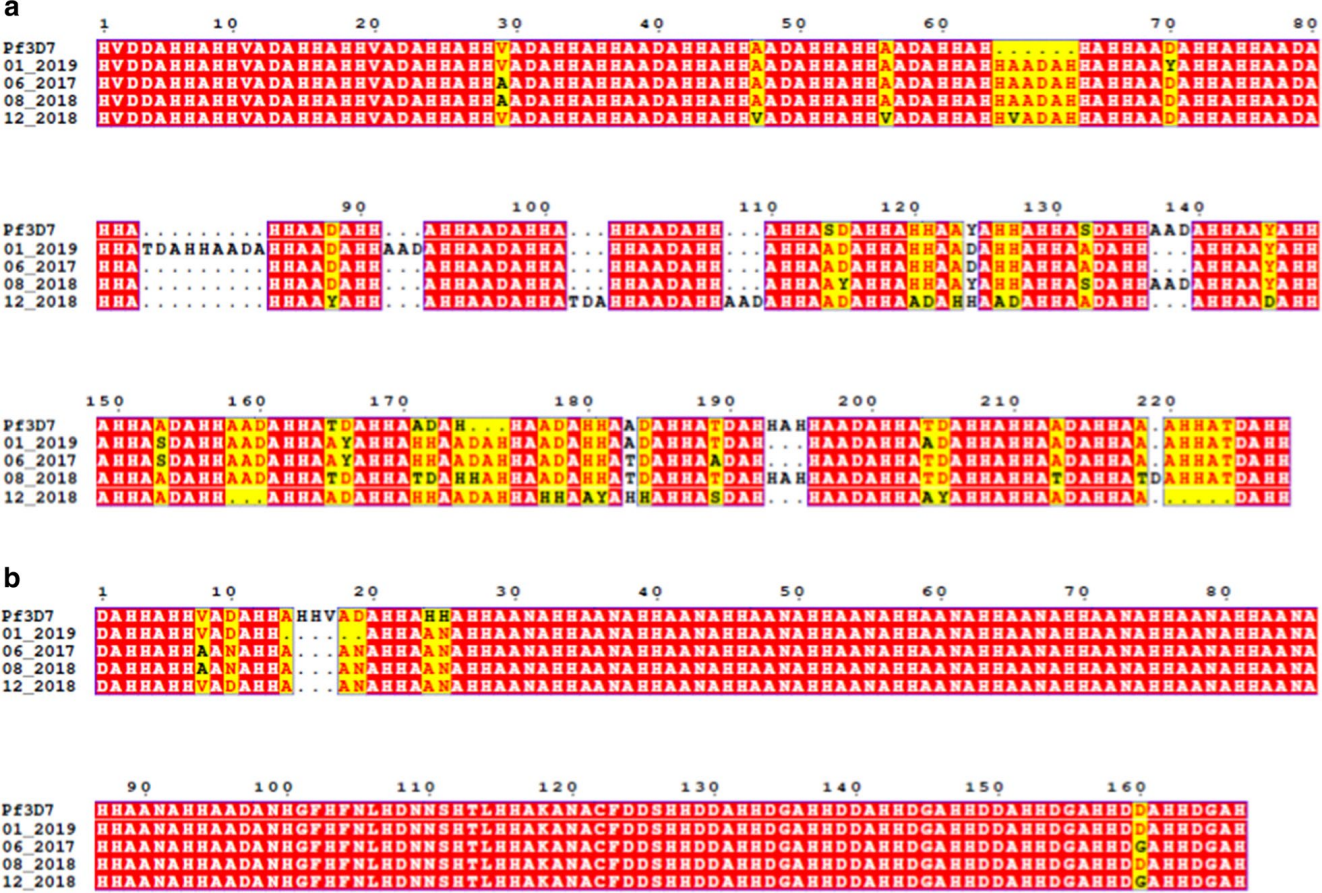
Amino acid alignments of *pfhrp2* (**a**) and *pfhrp3* (**b**) reveal genetic polymorphisms among *P. falciparum* isolates

Exon 2 of *pfhrp2* is the major source of repetitive motifs detected by PfHRP2-based RDTs; however, variations in the frequency of targeted repeats may influence accurate binding of specific antibodies [6]. As antigenic variants may have been the cause of these negative RDTs, amino acid sequences from exon 2 of *pfhrp2* (Fig. 4a) and *pfhrp3* (Fig. 4b), from the positive control and the three RDT-falsely negative samples, were aligned against the *P. falciparum* reference genome (Table 1, Fig. 4a). A high sequence polymorphism was found for PfHRP2. PfHRP2 contains repeated histidine and alanine motifs of which type 2 (AHHAHHAAD) and type 7 (AHHAAD) may be predictive of RDT sensitivity in low parasitaemia infections [11]. Here, according to the Baker’s model, predicting reactivity at parasites densities < 200 parasites/µL when the number of type 2 x type 7 repeats is > 43, only one isolate (08/2018) out of the three reported cases was predicted to escape detection (Table 2).

**Table 2:** *Pfhrp2* sequences of studied samples and predictive sensitivity of the antigenic test according to Baker’s model

*Pfhrp2* exon 2	Cameroun 06-2017	Ivory Coast 08-2018	Gabon 12-2018	Positive control 01-2019
Type 2 repeats AHHAHHAAD	15	13	9	11
Type 7 repeats AHHAAD	4	2	8	9
Baker’s score Type 2 × 7 repeats	60	26	72	99
Detected (Score > 43)	Yes	No	Yes	Yes

## Discussion

The aim of the present study was to investigate the prevalence and understanding the possible causes of RDT-negative *P. falciparum* cases over a long period during which two different diagnostic schemes were used at the Montpellier Academic Hospital (January 2006 to December 2018). Using the second diagnostic scheme, three cases of *P. falciparum* false-negative RDTs were detected, while no case had ever been reported when RDTs were combined solely with microscopy techniques. Amplifications of *pfhrp2* and *pfhrp3* in parasite isolates allowed ruling out gene deletion as the cause of non-detection by RDTs. Although this study presents some limitations due to its retrospective nature, it allowed reconsidering the ability of *Pf*HRP2-based RDTs to detect all *P. falciparum* infections in the laboratory and may thus be valuable for the community.

Various factors may influence the performances of *Pf*HRP2-based RDTs. First, results may be operator-dependent and rely on good product design and manufacturing quality [25]. All falsely negative RDTs analysed in this study were performed by well-trained operators. Two distinct brands were used, both known to give a panel detection score (*i.e.* the percentage of malaria samples in the panel giving a positive result) of more than 90% when tested on *P. falciparum* parasites at 200 parasites/µL [26]. Second, the level of parasitaemia is critical. Although some studies have reported RDTs failing to detect infections with high parasitaemia, the majority of these false-negative results occurred with low parasite densities, between 100 to 500 parasites/µL (reviewed in [10]). Detection rates of both RDT brands indeed decreased at these parasitaemia levels [26], e.g. 75% for SD Bioline Malaria Ag Pf/Pan^®^*vs* 100% for parasitaemia > 500 parasites/µL [27]. Here, three cases of false-negative RDT results were reported over 13 years: two displaying parasitaemia < 200/µL and one approximately 800 parasites/µL. If one may assume that the first two RDT failures were due to low parasite densities, the case from Gabon (12/2018) presenting 800 parasites/µL is intriguing.

Since the first demonstration of *P. falciparum* parasites lacking *pfhrp2* and *pfhrp3* genes in Peru [28], other studies, including African isolates, have reported deletions causing false-negative RDT results [29–34]. The World Health Organization recommends parasitological confirmation before treating patients with clinically suspected malaria [35]. In this context, parasites lacking *pfhrp2* or *pfhrp3* may have spread in a broader range of endemic regions, impairing clinical case management and control efforts for malaria elimination [36]. However, no deletion of *pfhrp2* nor *pfhrp3* genes was found in all three samples, consistent with the low prevalence of such deletions [37]. Alike previous studies reporting genetic diversity in field isolates from various geographical regions [10–12, 38–44] the *pfhrp2* gene was found highly polymorphic. A binary logistic model used to predict RDT detection sensitivity [11] revealed only one isolate (08/2018) at risk of non-detection. Yet, no statistical correlation between the frequency of repetitive epitopes and detection rates could be found in a previous study [39]. Moreover, genetic polymorphism does not appear to affect the detection of infections above 200 parasites/µL [11]. Taken together, genetic diversity of *pfhrp2* does not appear to be the cause of the increasing rate of *P. falciparum* negative RDT results in the laboratory. The possibility of low expression of HRP2/3 antigens cannot be ruled out in the absence of quantitative analysis at the protein level [45]. Anti-HRP2 antibodies binding to the circulating antigens may also reduce the diagnostic sensitivity of PfHRP2-based RDTs [46]. In addition, an infection with a mixture of HRP2-negative and HRP2-positive parasites (with a predominance of the first, the second being undetectable) remains a possibility.

The observed overall prevalence of falsely negative RDTs is low (0.67%), although similar to that found in France at a national scale between 2012 and 2017 (57/6118; 0.93%) (French National Reference Centre for Imported Malaria, S. Houzé, pers. commun.). It is interesting to relate the emergence of RDT false-negative results to the introduction of a novel strategy for the biological diagnosis of malaria in the laboratory. While no false-negative results had been identified over an 11-years period (2006–2016), all cases were reported between January 2017 and December 2018, *i.e.* after the implementation of a new malaria diagnostic scheme based upon a RDT in combination with a LAMP assay. A hypothesis to explain the emergence of falsely negative RDTs would be a reduction in quality of the tests at the stage of manufacture since 2017 but this has not been reported. One could also hypothesize that the introduction of a molecular method, more sensitive than microscopy and antigenic tests [14–18], has allowed detecting low parasitaemia infections for which the ‘classical’ RDT plus microscopy-based strategy may yield negative results. However, according to the records on the ‘pre-LAMP’ period, and given that the Academic Hospital deals with most malaria cases in the area, no patient with negative laboratory tests developed symptomatic malaria, suggesting that, if any, misdiagnosed patients have cured spontaneously. The use of microscopy for cross-checking RDT-negative results requires well-trained personnel and is time consuming, hence might not be suited for ruling out a diagnosis of malaria with very low parasite densities in a non-endemic setting [47]. The French National Authority for Health recommends to repeat screening tests after 24 h to 48 h in case of negative results in a suggestive clinical context [19]. Indeed, even low parasitaemia infections may result in symptomatic malaria in non-immune individuals; and misdiagnosis may delay the initiation of the treatment and sometimes result in a dramatic outcome [10].

## Conclusion

Through the analysis of *P. falciparum* RDT-negative results in a non-endemic setting, this study reviews the possible reasons for non-detection of HRP2/3 antigens and highlights the absence of gene deletion in *P. falciparum* infections diagnosed since 2006. It draws attention on the risk of missing malaria cases with low parasitaemia infections using the diagnostic strategy currently recommended by French authorities. In this context, this study may lay stepping-stones towards recommendations including molecular detection for malaria diagnosis in non-endemic countries.

## Data Availability

All data generated or analysed during this study are included in this published article.

## Abbreviations

RDT: Rapid diagnostic test
PfHRP2/3: Histidine-rich protein 2/3
LAMP: Loop-mediated isothermal amplification
pLDH: Pan lactate dehydrogenase
SPILF: French Infectious Diseases Society
WHO: World Health Organization
